# What are the changes in basketball shooting pattern and accuracy in National Basketball Association in the past decade?

**DOI:** 10.3389/fpsyg.2022.917980

**Published:** 2022-09-08

**Authors:** Feng Wang, Guohua Zheng

**Affiliations:** ^1^School of Journalism and Communication, Shanghai University of Sport, Shanghai, China; ^2^School of Physical Education, Xiangnan University, Chenzhou, China

**Keywords:** National Basketball Association, field-goal accuracy, basketball shooting pattern, performance evaluation, trend analysis

## Abstract

The main research question addressed in this study is if and how the shooting pattern and field-goal accuracy have changed in the NBA league in the past decade. This study analyzes NBA game data from the 2011–2012 regular season to the 2020–2021 season. Field goal attempts are grouped into five categories by the shooting distance. The Mann-Kendall trend test was employed to examine if changes are statistically significant (*p* < 0.05) over the years. Sixteen equal segments in one basketball game, each with 3 min, were analyzed to examine the shooting pattern in different game segments. Results reveal an increasing trend in the percentage of 3-pointer shooting, which has nearly doubled from 22 to 39%. Meanwhile, the percentage of field goals within the range of 16–24 ft has decreased from 20 to 10%. Field-goal accuracy has shown an increasing trend for all shooting distances except for the 3-pointer shooting. The second and fourth 3-min within each quarter have the highest number of field goals. The first quarter has a higher shooting accuracy than the rest three quarters. In addition, results reveal that the last 3-min in each quarter has the lowest shooting accuracy. Reasons for the patterns of field goals in different segments are discussed from the perspective of game rule changes, the fatigue effects, and coaches’ game strategies. The reasons for changes in activity level and performance in different quarters are also discussed. This study offers new insights into the changes in basketball shooting patterns and accuracy in NBA games in the past decade. Practical meanings of this study for basketball players, coaches, and sports psychologists, as well as the strength and limitations of this study, are discussed.

## Introduction

As one of the sports with a large population base globally, basketball continues to gain popularity (Gulak-Lipka, 2020). The popularity of the National Basketball Association (NBA) basketball games stems from the athleticism of professional players, the excitement such competitive games offer to the fans, and the successful business operation of the league (Nourayi, 2019). During the regular season, each team aims to win many games as possible to enhance its chance to compete in the playoffs.

Multiple factors affect a team’s performance during a regular NBA season, e.g., coaches’ tactics, basketball players’ fitness, and game location (home or away) (Zhang et al., 2019; Huyghe et al., 2022). Those factors can be divided into two categories, namely, player performance and external factors. The former includes players’ shooting accuracy, turnovers, rebounding, and free throws. External factors include game locations and coaches’ tactics (Wang and Zheng, 2022). Teramoto and Cross (2010) discussed the relative importance of performance factors in winning basketball games using 1999–2009 NBA data. Their results indicated that both offensive and defensive goal percentages are the most critical aspects of the game in the regular season. In addition, efficient offense and defense are essential to success in the regular season. Mikolajec et al. (2013) studied eight seasons (2003–2011) of NBA games and identified several factors that determine team performance, including offensive efficiency, 3rd quarter points per game, average fouls, and average steals. The concept of momentum effect has been used to describe the situation in which a team has a higher probability of winning basketball games had the team been playing well in the last few games. Using data over three NBA seasons (2007–2009), Arkes and Martinez (2011) found that greater success in the past few games leads to a higher probability of winning the next game. Likewise, poor play over the past few games leads to a lower probability of winning the next game.

Among the many performance indicators, the shooting accuracy of field goals in basketball games is found as a critical factor one that determines the outcome of basketball games in different levels of professional leagues (Akers et al., 1992; García et al., 2013; Puente et al., 2015; Çene, 2018). The accuracy of field goals varies depending on shooting distances, and changes in the accuracy of field goals from the same shooting distance over the years have been reported (Wang and Zheng, 2022).

Changes have also been made to NBA basketball games in the past years (Nourayi, 2019). Most rule changes introduced in the NBA aim to keep enhancing the pace, speed, and flow of basketball games. For instance, basketball teams were allowed to have only two, instead of three, time-outs in the final 3 min of the games starting from the 2017–2018 season. Responding to rule changes, Professional players changed shooting style or spot selection in basketball games. Mandić et al. (2019) analyzed the 2000–2017 NBA data and found an increasing pace of basketball games, owing to a series of changes to off-ball fouls, clear-path fouls, and similar changes which aimed to quicken the flow of the game. Kilcoyne (2020) found a decline in the mid-range jump shot, defined as field goals with a shooting distance in the range of 8–24 ft, by analyzing NBA data from the 2005–2006 season through 2018–2019. The defined range between 8 and 24 ft in Kilcoyne (2020) seems not narrow enough to examine if the decline occurred in the shooting distance of 8–16 ft or 16–24 ft. An analysis of a rich dataset is needed to provide insights into whether mid-range accuracy decline occurred in the shooting distance of 8–16 ft or 16–24 ft. In addition, there lacks an examination of field-goal accuracy changes in the restricted area. To fill the gap, this study comprehensively analyzes changes in the shooting style and field goal accuracy in the NBA league.

Specifically, this study aims to understand what percentages of field goals are attempted from different shooting distances and if such percentage values change over the years. Similar questions apply to the accuracy of field goals from different shooting distances. What is the field-goal accuracy from different shooting distances? Does field-goal accuracy change over the year? This study’s experimental hypothesis is that the percentage of field-goal from different shooting distances and field-goal accuracy from different distances have not changed significantly over the past decade. In addition, this study aims to answer questions such as how the total number of field goals is distributed over different segments of a basketball game and the success rate of field goals in each segment of a typical NBA game. Rather than focusing on individual basketball players, this study examines relevant statistics calculated from all basketball players in the NBA league since the objective is to investigate potential trends in game-related statistics over the past decade.

## Materials and methods

### Sample and procedures

Due to the availability of sophisticated technology, a mass of data is generated for each NBA game, including the location of the field goals attempted and made, the time of occurrence of each field goal, and the type of field goals, i.e., 2-point field-goal or 3-point field-goal. NBA game data in the past decade, starting from the 2011–2012 season to the 2020–2021 season, were obtained in this study.^1^ Each of the 30 NBA teams has played 82 games in a regular season in the past decade, with only two exceptions. The total number of basketball games is 1,230. One exception is that the 2011–2012 regular season was shortened from 82 games per team to 66 due to nearly 2 months of inactivity. The other exception is the 2019–2020 regular season affected by the COVID pandemic, ending with a shortened season of 1,059 games. The 2020–2021 season was again affected by the COVID pandemic, in which each team played 72 games, resulting in 1,080 games in the regular season.

To facilitate data retrieving, the NBA shot chart application programming interface (API) was used to download data from the NBA’s website. The python code that was used to collect NBA game data is provided in Supplementary material. Field goal attempts are grouped into five categories by the shooting distance. The first category is field goals with a shooting distance of less than 8 ft and within the restricted area. The second category is field goals with a shooting distance less than 8 ft but not in the restricted area. The third category has a shooting distance between 8 and 16 ft. The fourth category falls into the range of 16–24 ft, and the last group is 24 ft and above.

**FIGURE 1:**
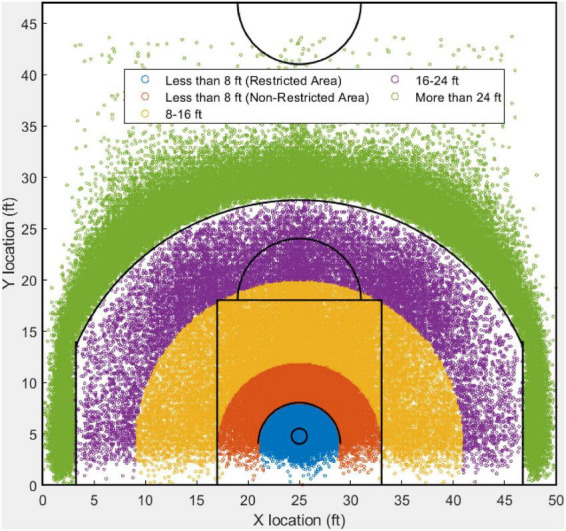
An illustrative example of field goals from five different shooting distances based on the NBA regular season 2020–2021.

Figure 1 illustrates the field goals for the 2020–2021 NBA season. All field goals are colored based on the group. Within the 1,080 games in the regular season, there were in total 190,983 field goal attempts. Field goals from different shooting zones are represented in distinct colors.

#### Measures

A few notations are introduced to describe the evaluation metrics used in this study. Let *N_*d*,t_* denote the number of field goals attempted from different shooting distances *d* (*d* = 1,2,…,5 representing zones with an increasing shooting distance) at the *i*th regular season (*t* = 1,2,…, 10 representing different NBA seasons in time ascending order); *M_*d*,t_* represents the number of field goals made from different shooting distances s at the *t*th regular season. The total number of games in each season is denoted as *G*_*t*_.

The total number of field goals per game in each regular season, denoted as $\bar{N_{t}}$, reflects the flow of the games, which can be calculated in Equation (1).


(1) $$\bar{N_{t}}=\frac{\sum_{d=1}^{5}N_{d,t}}{G_{t}}$$

The percentage of field goal attempts from different shooting distances in each season, denoted as *r*_*d*,*t*_, can be calculated using Equation (2).


(2) $$r_{d,t}=\frac{N_{d,t}}{\sum_{d=1}^{5}N_{d,t}}\times 100\%$$

The accuracy of field goals (in percentage) from different shooting distances in each NBA regular season can be calculated using Equation (3).


(3) $$p_{d,t}=\frac{M_{d,t}}{N_{d,t}}\times 100\%$$

To further examine the distribution of field goals in different segments of the games. Each quarter of a game is divided into four equal segments, each with a game time of 3 min. A notation with two numbers connecting through a line, e.g., 1–1, is introduced to represent a corresponding segment in a quarter of a basketball game. The first number denotes the quarter, and the second number represents the segment. For instance, 2–3 denotes the third segment (min 6–9) of the second quarter of a game. Let *N_*s*,t_* denote the number of field goals attempted from different segments *s* (*s* = 1,2,…,16 representing different segments in a game) at the *t*th regular season (*i* = 1,2,…, 10 representing different NBA seasons in time ascending order); *M_*s*,t_* represents the number of field goals made from the *s*th segment (*s* = 1,2,…,16) at the *t*th regular season.

The percentage of field goal attempts from different shooting segments in a basketball game in each season, denoted as *r*_*s*,*t*_, can be calculated using Equation (4).


(4) $$r_{s,t}=\frac{N_{s,t}}{\sum_{s=1}^{16}N_{s,t}}\times 100\%$$

The accuracy of field goals (in percentage) from different segments of a basketball game in each NBA regular season can be calculated using Equation (5).


(5) $$p_{s,t}=\frac{M_{s,t}}{N_{s,t}}\times 100\%$$

#### Statistical analysis

The Mann-Kendall test, which is often referred to as the M-K trend test, is applied to detect if any trend exists in the performance metrics over the past decade. The M-K trend test is a rank-based non-parametric test with wide applications. It is applicable only when all the observations in a time series are serially independent. To conduct an M-K trend test, the data were first ranked according to time, and then each data point was treated as a reference data point and was compared to all of the data points that followed in time. Details of the method are omitted to save space, and interested readers are referred to other studies (Mann, 1945; Kendall, 1975; Liu et al., 2013). The null hypothesis of an M-K trend test is that no increasing or decreasing trend exists in the data. A significance level of 0.05 was employed in this study to detect if the null hypothesis could be rejected. When the *p*-value is larger than 0.05, the null hypothesis cannot be rejected. Only a test with a *p*-value less than 0.05 indicates a statistically significant trend exhibited in the data. The software Matlab version 2020a was used in data analysis in this study.

## Results

**FIGURE 2:**
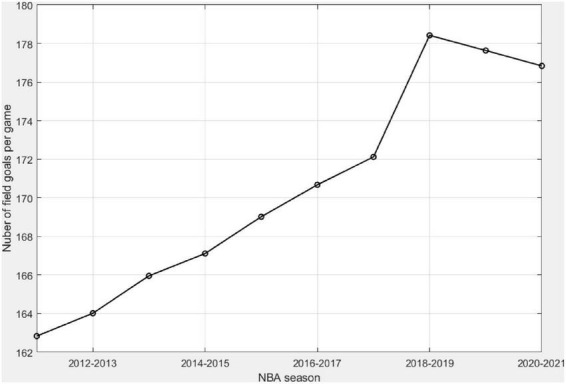
Number of field goals attempted from all shooting distances in a game for each season between the 2011–2012 regular season and the 2020–2021 regular season.

Figure 2 shows the average number of field goals, $\bar{N_{t}}$, aggregated from all various shooting distances per basketball game in different NBA regular seasons in the past decade. It was about 163 field goals in the 2011–2011 season and jumped to 178 in the 2018–2019 season. While there seemed to be a slight decrease in the last two seasons in Figure 1, those numbers are still much higher than earlier seasons in the 2010s. Note that the increase of field goals per game seems steady from the 2011–2012 season to the 2017–2018 season, with an increase of 1–2 field goals per game each year. However, the magnitude of changes from the 2017–2018 season to the 2018–2019 regular season was larger. The *p*-value of the M-K trend test for $\bar{N_{t}}$ is much less than 0.05. Hence, an increasing trend of statistical significance is identified (Table 1).

**TABLE 1:** The *P*-values of the Mann-Kendall trend test for different variables in this study.

Variable name	Variable explanation	*P*-value	Trend
$\bar{N_{t}}$	Number of field goals per game	*P* < 0.001	+
*r*_1,*t*_	Percentage of field goals in the restricted area	0.150	
*r*_2,*t*_	Percentage of field goals with a shooting distance < 8 ft and outside the restricted area	0.470	
*r*_3,*t*_	Percentage of field goals with a shooting distance of 8–16 ft	0.007	_
*r*_4,*t*_	Percentage of field goals with a shooting distance of 16–24 ft	*p* < 0.001	_
*r*_5,*t*_	Percentage of field goals with a shooting distance of over 24 ft	*p* < 0.001	+
*p*_1,*t*_	Accuracy of field goals in the restricted area	0.012	+
*p*_2,*t*_	Accuracy of field goals with shooting distance < 8 ft and outside the restricted area	0.020	+
*p*_3,*t*_	Accuracy of field goals with a shooting distance of 8–16 ft	*p* < 0.001	+
*p*_4,*t*_	Accuracy of field goals with a shooting distance of 16–24 ft	*p* < 0.001	+
*p*_5,*t*_	Accuracy of field goals with a shooting distance of over 24 ft	0.150	
rs,t∼s,t	The median of field goal percentages in different segments of a basketball game	0.043	_
ps,t∼s,t	The median of field goal accuracy in different segments of a basketball game	0.034	_

The plus sign (+) indicates an increasing trend, while the minus sign (–) indicates a decreasing trend.

**FIGURE 3:**
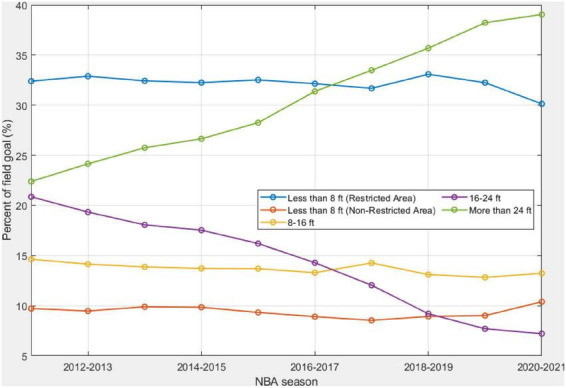
Changes in the percentage of field goals from each of the five shooting distances in the past decade.

Figure 3 depicts the percentage of field goals from different shooting distances in each NBA regular season. This reflects changes in the spot selection of professional basketball players. The *p*-values of the M-K trend test for *r*_*d*,*t*_ are statistically significant except for the field goals with a shooting distance of less than 8 ft, as shown in Table 1. There are a few observations from Figure 3. First, the percentage of field goals over 24 ft, i.e., 3-pointers, has been increasing from 22% in the 2011–2012 season to 39% in the 2021–2022 season. This has almost doubled and is increased by nearly 1.85% each year. Second, the percentage of field goals within the range of 16–24 ft has shown a steady decrease from 21% in the 2011–2012 season to nearly 7.2% in the 2020–2021 season. Third, the ranking of preferred shooting distances has changed because of the preference switch in the long-range shots over 24 ft and mid-range shots within the distance of 16–24 ft. At the beginning of the 2010s, the field goal within the restricted area was the top selection of shooting zones with a percentage value of 32.4%. Field goals with a shooting distance less than 8 ft but outside the restricted area are the least favorable shooting zone, with a percentage value of 9.73%. The ranking of the favorable shooting zones was the same until the 2016–2017 regular season. With the continuing increase of long-range field goals over 24 ft, the ranking started to change in the 2017–2018 season. For the 2020–2021 season, long-range shots are the most favorable, and mid-range shots within the distance of 16–24 ft are the least favorable shooting zone. The last but not the least observation from Figure 3 is that the variance in the percentage values of the five shooting distances is larger at the end of the 2010s, compared to the earlier years in the 2010s. For instance, the standard deviation for the 2011–2012 season is 8.6%; and it is 13.8% for the 2020–2021 season.

**FIGURE 4:**
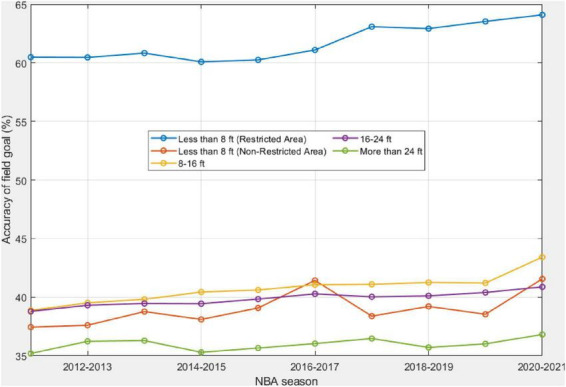
Changes in the accuracy of field goals from each of the five shooting distances in the past decade.

Figure 4 shows the accuracy of field goals from different shooting zones, *p*_*d*,*t*_, over the past decade. As expected, field goals within the restricted area have the highest accuracy, which is higher than 60% for all the years. The long-range shots have the lowest accuracy, within the range of 35–36%. The accuracy of field goals within the paint, with a distance less than 8 ft outside the restricted area, is lower than mid-range field goals. Another observation from Figure 4 is that an increasing trend exists for all except the long-range field goals over 24 ft, although the increased percentages are different. The substantial increases in field goal accuracy in the restricted area indicate that big players have been playing efficiently in attacking the basket.

The *p*-values of the M-K trend test for *p*_*d*,*t*_ are statistically significant except for the long-range field goals over 24 ft. Hence, an increasing trend of statistical significance is identified for field goals in the remaining categories, as shown in Table 1.

**FIGURE 5:**
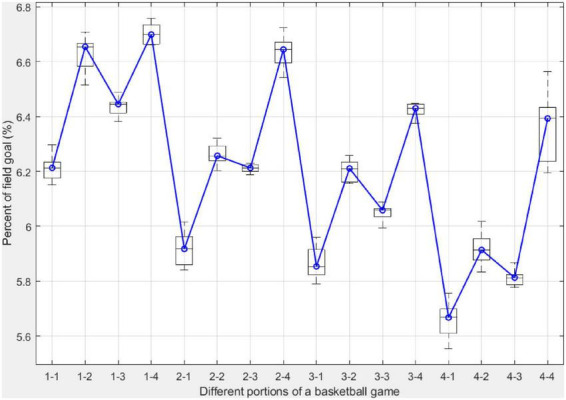
The percentage of the number of field goals from different 3-min segments in a basketball in the past decade. Each boxplot is comprised of 10 values, each corresponding to one NBA regular season in this study. The blue line connects the median of the boxplots.

Figure 5 shows the percentage of field goals attempted from different segments of a basketball game. Each boxplot of a segment is comprised of ten values, each representing the percentage of field goal attempts from the corresponding segment in one NBA regular season. The blue line in Figure 5 connects the median of all boxplots rs,t∼s,t. The *p*-value of the Mann-Kendall test for rs,t∼s,t is statistically significant, indicating a decreasing trend. In addition, there are a couple of observations from Figure 5. First, the number of field goal attempts from each of the four segments in the first quarter is higher than those in the corresponding segment in the remaining three quarters. For instance, the median value is 6.45% for the third segment in the first quarter, which is higher than the median values of segments 2–3, 3–3, and 4–3. Similarly, the percentage of field goal attempts in corresponding segments in the first quarter is higher than the same segment in the other quarters. Another interesting observation is the pattern of field goal attempts within a quarter. The first segment in each quarter has a lower percentage of field goals compared to the last segment in the same quarter.

**FIGURE 6:**
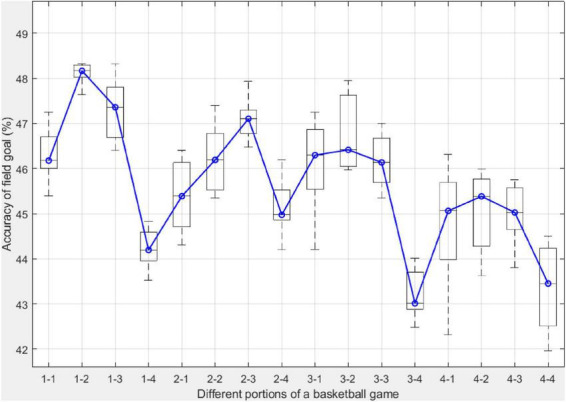
The accuracy of field goals from different 3-min segments in a basketball in the past decade. Each boxplot is comprised of 10 values, each corresponding to one NBA regular season in this study. The blue line connects the median of the boxplots.

Similarly, Figure 6 shows the accuracy of field goals attempted from different segments of a basketball game. Each boxplot of a segment is comprised of ten values, each representing the accuracy of field goal attempts from the corresponding segment in one NBA regular season. The blue line in Figure 6 connects the median of all boxplots ps,t∼s,t. Overall, field goal accuracy decreases when the basketball game proceeds, evidenced by the *p*-value of the Mann-Kendall test ps,t∼s,t. In addition, All segments except the fourth segment in the first quarter have higher accuracy than the corresponding segment in the other three quarters.

## Discussion

This study aims to understand the percentages of field goals attempted from different shooting distances and if such percentage values have changed in the last decade. Analyses were conducted to examine the field-goal accuracy from different shooting distances and if field goal accuracy has changed over the years. In addition, efforts were also made to understand the percentage and accuracy of field goals in different segments of a basketball game. The main results can be summarized below:

• The number of field goals per game has increased from 163 in the 2011–2011 season to 177 in the 2020–2021 season, with a
• The percentage of field goals over 24 ft has almost doubled, increasing from 22% in the 2011–2012 season to 39% in the 2021–2022 season.
• The percentage of field goals within the range of 16–24 ft has shown a steady decrease from 21% in the 2011–2012 season to nearly 7.2% in the 2020–2021 season.
• An increasing trend exists in the field goal accuracy for all except the long-range field goals over 24 ft.
• The number of field goal attempts and field goal accuracy from each of the four segments in the first quarter is higher than those in the corresponding segment in the remaining three quarters.

This section discusses major factors that potentially lead to those findings presented in the results section.

### The increasing pace of National Basketball Association games

As shown in Figure 2, there has been an increasing pace and flow in the NBA games in the past decade, reflecting changes in the game rules and the changes in playing style adopted by professional teams. The finding is consistent with Mandić et al. (2019), in which authors analyzed the 2000–2017 NBA data and found an increasing pace of basketball games, which can be explained by a series of changes to game rules. The relatively significant increase in the number of field goals per game from the 2017–2018 season to the 2018–2019 regular season is probably due to the rule changes made to clear-path fouls. A field-goal attempt with a similar offense and defense position could be counted as a clear-path foul in the prior seasons but not in the 2018–2019 season. This has led to increased field goals in the 2018–2019 season.

### The main driver for 3-pointer increasing

The increase of 3-pointer in the shot distribution is consistent with findings from other studies (Kilcoyne, 2020; Freitas, 2021). The NBA league moved the 3-point line in the 1994–1995 season to enhance scoring across the league. Although the 3-point line was moved back to its original distance after a 3-year experiment, there is a long-lasting influence on shot selection. Many coaches in the league incorporated the three-point field goal into offensive strategies. Professional players with higher accuracy from the long-distance have become more commonplace across the league. Another driving factor contributing to the switch of spot selection between the long-distance and 16–24 ft is that players are primarily encouraged to drive to the basket or shoot a three-pointer (Kilcoyne, 2020). This has resulted in the continuing decline of the mid-range shot with a shooting distance of 16–24 ft since the 2011–12 season. Kilcoyne (2020) also reveals the decline in the mid-range field goals, but the author did not differentiate shooting distances between 8–16 ft and 16–24 ft. Our study finds a slight change in the percentage of field goals within the 8–16 ft. Meanwhile, a steady decline over the past decade has been found in the field goal attempt in the 16–24 ft. Kilcoyne (2020) lumped all 2-point field goals, ignoring the different shooting distances within the 3-point line, in analyzing the percentage of 2-point and 3-point field goals.

### The main driver for increased field-goal accuracy

Increases in the accuracy of the 2-point field goals may be due to different reasons. First, several rule changes occurred, such as banning hand-checking, in earlier years prior to the 2010s, which encouraged offensive players more freedom to score at a higher efficiency. As another example, a rule change to the clear-path fouls (Mandić et al., 2019) has also improved field goal attempts and accuracy. Second, similar to the increased percentage of field goals with a shooting distance over 24 ft, professional players are encouraged to drive to the basket. Hence, driving layup shot or dunk shot has become more commonplace. Third, with the wide application of data analytics in coaching and game strategy design, coaches and players can design tailored game strategies and make wiser decisions regarding shot spot selection during the game. This would lead to enhanced field goal accuracy.

### Activity level and performance in different quarters

The fatigue effect and game strategy are the two main external factors that the percentage and accuracy of field goals in different segments of a basketball game. The fatigue effect refers to the fact that with the progression of the game, intense offense and defense burn an increased amount of energy for basketball players, often resulting in reduced activity and performance (Kordyaka et al., 2022). Another evidence of the fatigue effect is that although the fourth segment has the highest percentage of field goal attempts within its corresponding quarter, the field goal accuracy is the lowest. Figure 5 also reveals the pattern of field goal attempts within a quarter. The first segment in each quarter has a lower percentage of field goals compared to the last segment in the same quarter. This is largely explained by game strategies adopted by the coaches. Basketball teams emphasize defense at the beginning of each quarter. In the last 3 min of each quarter, when strategic substitution often occurs, basketball teams play more offensive in attacking the basket, taking the opportunity to come back from the game or maintain the advantage in the score. Within a quarter, the first segment has a lower accuracy than the second. It then decreases in the third and fourth segments while the second quarter is the only exception. This is probably due to the contribution of field goals in the restricted area, which has the highest success rate.

### Strengths and limitations

There are a few strengths associated with this study. First, it uses a data extraction technique to obtain a rich dataset that allows an examination of field goals from different shooting distances and various segments of a basketball game. Second, it is the first kind of analysis that examines the shooting pattern, field goal accuracy over the past decade, and activity level and performance in different quarters of the NBA games. Results from this study are consistent with the existing literature but offer additional insights. In addition, study results have practical implications for basketball coaches. For instance, field-goal accuracy from different distances in the last 3–5 years allows coaches to compare an individual team’s performance to the average performance of all the teams in the league. This facilitates basketball coaches to design targeted training to improve players’ field-goal accuracy, e.g., focusing on 8–16 ft field-goal or field-goal with a shooting distance of more than 24 ft. Moreover, results from this study have practical meanings for sports psychologists. For instance, athletes may have pressure from their expectation of field goal accuracy during crunch time in a basketball game. Understanding the changes in performance and activity level when a basketball proceeds, sports psychologists position themselves better to help athletes cope with the pressure of competition. As another example, sports psychologists need to have a good understanding of field goal accuracy in recent years to help athletes to keep motivated and shape their mental toughness to continue an exercise program to improve their field goal accuracy.

Nevertheless, there are also limitations of this study. Although the shooting zones are grouped by shooting distance, each field goal within a certain shooting distance, e.g., 8–16 ft, is not strictly the same. The shooting time within the 24-s clock, defense intensity, and shooting habit of an individual player could affect the accuracy of a field goal. This study does not consider such factors affecting the accuracy due to data unavailability.

## Conclusion

This study analyzed NBA games in the regular season for the past decade to examine the potential changes in the percentage and accuracy of field goals from distinct shooting distances. Results reveal that there has been an increasing number of field goals per game. In addition, there are significant changes in the shooting distances where players choose to shoot the basketball. Field-goal accuracy for all the shooting zones has shown an increasing trend except for the 3-pointer shooting. The driving factors that lead to such changes are discussed in this paper. This study also analyzes the temporal pattern of the percentage and accuracy of field goals at different segments of the game. The two major contributing factors, i.e., fatigue effect and game strategy, are discussed. Results from this study, on the one hand, are in line with findings in the literature. On the other hand, this study offers new insights into the understanding of changes in basketball shooting patterns and accuracy in NBA games in the past decade. Practically, an enhanced understanding of those changes allows professional athletes and coaches to design targeted training and game strategies when competing in the NBA.

## Data availability statement

The original contributions presented in this study are included in the article/Supplementary material, further inquiries can be directed to the corresponding author.

## Author contributions

FW and GZ originated the project and performed statistical analysis. FW processed and analyzed the data. Both authors discussed the results and actively contributed to the final manuscript.

## Footnotes

1
https://www.nba.com/stats/
