# Supplementary material for: What are the changes in basketball shooting pattern and accuracy in National Basketball Association in the past decade?
Source: Front Psychol. 2022 Sep 8;13:917980. doi: 10.3389/fpsyg.2022.917980 (PMC9494817; doi:10.3389/fpsyg.2022.917980)
Supplement: Supplementary file 1 [file Data_Sheet_1.pdf]

```python
# import nba API
from nba_api.stats.endpoints import shotchartdetail
from nba_api.stats.static import players
# import tools for data formating
import json
import pandas as pd
# get data from NBA api
response = shotchartdetail.ShotChartDetail(
    context_measure_simple='FGA',
    team_id=0,
    player_id= 0,
    season_nullable='2018-19',
    season_type_all_star='Regular Season'
)

content = json.loads(response.get_json())

# transform contents into dataframe
results = content['resultSets'][0]
headers = results['headers']
rows = results['rowSet']
df = pd.DataFrame(rows)
df.columns = headers

# save data
df.to_csv('chart_data_2018_2019_playoffs.csv')
```
